# Influenza Surveillance among Outpatients and Inpatients in Morocco, 1996–2009

**DOI:** 10.1371/journal.pone.0024579

**Published:** 2011-09-08

**Authors:** Amal Barakat, Hassan Ihazmad, Samira Benkaroum, Imad Cherkaoui, Abderahman Benmamoun, Mohammed Youbi, Rajae El Aouad

**Affiliations:** 1 Centre National de Référence de la Grippe, Institut National d'Hygiène, Ministère de la Santé, Rabat, Morocco; 2 Institut National d'Hygiène, Ministère de la Santé, Rabat, Morocco; 3 Direction de l'épidémiologie et de Lutte contre les Maladies, Ministère de la Santé, Rabat, Morocco; University of Liverpool, United Kingdom

## Abstract

**Background:**

There is limited information about the epidemiology of influenza in Africa. We describe the epidemiology and seasonality of influenza in Morocco from 1996 to 2009 with particular emphasis on the 2007–2008 and 2008–2009 influenza seasons. Successes and challenges of the enhanced surveillance system introduced in 2007 are also discussed.

**Methods:**

Virologic sentinel surveillance for influenza virus was initiated in Morocco in 1996 using a network of private practitioners that collected oro-pharyngeal and naso-pharyngeal swabs from outpatients presenting with influenza-like-illness (ILI). The surveillance network expanded over the years to include inpatients presenting with severe acute respiratory illness (SARI) at hospitals and syndromic surveillance for ILI and acute respiratory infection (ARI). Respiratory samples and structured questionnaires were collected from eligible patients, and samples were tested by immunofluorescence assays and by viral isolation for influenza viruses.

**Results:**

We obtained a total of 6465 respiratory specimens during 1996 to 2009, of which, 3102 were collected during 2007–2009. Of those, 2249 (72%) were from patients with ILI, and 853 (27%) were from patients with SARI. Among the 3,102 patients, 98 (3%) had laboratory-confirmed influenza, of whom, 85 (87%) had ILI and 13 (13%) had SARI. Among ILI patients, the highest proportion of laboratory-confirmed influenza occurred in children less than 5 years of age (3/169; 2% during 2007–2008 and 23/271; 9% during 2008–2009) and patients 25–59 years of age (8/440; 2% during 2007–2009 and 21/483; 4% during 2008–2009). All SARI patients with influenza were less than 14 years of age. During all surveillance years, influenza virus circulation was seasonal with peak circulation during the winter months of October through April.

**Conclusion:**

Influenza results in both mild and severe respiratory infections in Morocco, and accounted for a large proportion of all hospitalizations for severe respiratory illness among children 5 years of age and younger.

## Introduction

Since 1948, the World Health Organization (WHO) Global Influenza Surveillance Network has provided a platform to monitor circulating influenza types and subtypes to make informed decisions about the composition of influenza vaccine [1]. In 2001, the WHO formulated a Global Agenda for Influenza Surveillance and Control which highlighted priority strategies to reduce morbidity and mortality from annual influenza epidemics and prepare for influenza pandemics. These strategies include: strengthening surveillance, improving knowledge of burden of influenza, increasing vaccine usage, and accelerating pandemic preparedness [2]. Influenza sentinel surveillance, which includes the collection of epidemiological and virological data, provides a tool to monitor the circulating types and subtypes of influenza to assess the burden of influenza in the community and to rapidly detect the appearance of novel influenza subtypes in the human population. These data can be used to understand geographic, temporal, and biologic differences in circulating influenza strains and to monitor for the emergence and evolution of pandemic strains [3]–[5].

The prevalence and burden of influenza are well documented in the temperate countries of the northern and southern hemispheres [5]–[13]. In contrast much less is known about the epidemiology, burden and patterns of transmission of influenza in tropical countries and even less in the African continent. A recent systematic review of available data from Africa emphasized that data from Africa are insufficient to allow most countries to prioritize strategies for influenza prevention and control [14], [15]. Identified data gaps in Africa include temporal patterns of influenza virus circulation, the incidence of influenza-associated outpatient visits, hospitalizations, and mortality for all ages, and the contribution of influenza to the overall burden of respiratory illness hospitalizations among adults and children. In Morocco, influenza sentinel surveillance was established in 1996 [16] and was expanded starting in 2007 in response to the emergence of the highly pathogenic avian influenza A (H5N1) threat. Using influenza surveillance data from Morocco from 1996–2009, we describe the seasonality of influenza virus circulation, identify age groups accounting for a large proportion of influenza-associated outpatient visits and hospitalizations, and discuss the successes and challenges of expanding influenza surveillance during 2007–2009.

## Methods

### Study design and setting

Morocco is located in the temperate zone of Northern Africa and encompasses Atlantic and Mediterranean coast lines as well as mountainous and desert areas. The country is divided into 16 administrative regions and 81 prefectures and provinces. Each administrative region has a tertiary care regional referral hospital that is linked to provincial hospitals and in turn to the nationwide system of health centers. Health care in Morocco is provided by public hospitals and health centers as well as private clinics. Though people may utilize medical services in either sector, in general, medical services provided through the public sector are less expensive than those provided through the private sector. According to the 2007 statistics of the Ministry of Health of Morocco, approximately 50% of the population receives health care through the public sector [17]. Influenza vaccine is not provided by the public sector. However, seasonal influenza vaccine is available from private pharmacies.

### Influenza sentinel surveillance during 1996–2003

During 1996–2003, the National Influenza Center (NIC) situated at the National Institute of Hygiene conducted virological surveillance using a network of volunteer private practitioners that identified patients with influenza-like illness (ILI) or acute respiratory illness (ARI) from patients presenting at the sentinel clinics and collected specimens for influenza testing and basic demographic data from a subset of patients with ILI each year during October through April. ILI was defined as an outpatient with fever (≥38°C) and cough or sore throat with onset less than five days prior to presentation in the absence of a specific diagnosis. ARI was defined as an outpatient with sudden onset of respiratory signs including cough, difficulty breathing, rhinitis or coryza and general symptoms such as fever, headache, fatigue, and myalgia less than five days prior to presentation.

### Influenza sentinel surveillance during 2004–2006

In 2004, the Epidemiology Department at the Ministry of Health expanded the existing system through the implementation of syndromic surveillance for ARI and ILI in the 16 administrative regions of the country using a network of 375 public clinics covering all provinces and prefectures and a population of 12 million people. Aggregated numbers of ARI and ILI cases were collected.

### Influenza sentinel surveillance during 2007–2009

Starting in 2007, the system was further enhanced through the collection of virological and epidemiological data from both the private practitioner and public clinic networks. One health centre in each region collected samples from the first 5 ILI cases identified each day. In addition, the 110 private practitioners participating in the virological sentinel surveillance network began providing clinical and demographic data for all ARI and ILI cases during October to April and began reporting the total number of consultations by week.

Furthermore, surveillance for severe acute respiratory illness (SARI) was established in 14 of the 16 regions of the country. In patients five years of age and older, SARI was defined as a hospitalized patient with fever (>38°C), cough, and shortness of breath or difficulty breathing with duration of illness less than seven days. In patients two months through five years of age, SARI was defined as a child hospitalized for cough or difficulty breathing, with or without wheezing and stridor in a calm child, or chest indrawing. In patients 1 week through 2 months of age, SARI was defined as hospitalization for a respiratory illness associated with one of the following signs: convulsions, rapid breathing (≥60 breaths per minute in a calm infant), chest indrawing, nasal flaring, grunting, lethargy or unconsciousness, decreased movement, fever (>38°C or warm to touch), or hypothermia (<36°C or cold to the touch). SARI cases were identified from patients hospitalized in the pediatrics, pneumonology, and internal medicine units in the 14 regional hospitals. All SARI cases were eligible for enrollment. Data on gender, age, clinical symptoms, treatment, influenza vaccination status and travel history in the 7 days prior to symptom onset were collected from all ILI and SARI patients that were enrolled in the virological surveillance system using a standard case report form.

During 2007–2008, a laboratory network of 14 regional laboratories supervised by the NIC was established. Two additional laboratories joined the network during 2009–2010.

### Human Subjects Review

All surveillance protocols were reviewed and approved by the Ethic Committee of the Morocco Ministry of Health.

### Sample collection and laboratory procedures

During 1996–2009, oro-pharyngeal and naso-pharyngeal swabs were collected from ILI and SARI patients enrolled under the virological surveillance system and placed in cryovials containing 2 ml of virus transport medium. Oro-pharyngeal and naso-pharyngeal swabs collected from the same patient were placed in one cryovial, stored at 4°C at the sentinel site, and transported daily to the NIC or the Regional Reference Laboratories. For transportation, the specimens were packaged using a standard triple packaging system at the sentinel sites and were transported in cool boxes.

Until 2007, all ILI samples collected under the public and private sentinel surveillance network were sent to the NIC for testing. Starting in 2007, SARI and ILI samples collected under the public sentinel surveillance network were sent to the Regional Reference Laboratories. All specimens were tested for influenza A and B viruses, adenovirus, parainfluenza 1–3 and respiratory syncytial virus using commercial indirect immunofluorescence kits (VRK Bartels). All SARI samples and influenza-positive ILI samples were further sent to the NIC for confirmation of influenza results by virus isolation using Madin Darby Canine Kidney (MDCK) cell lines and haemagglutination inhibition assay for typing and subtyping of positive influenza samples using the standardized WHO reagents and protocols. Specimens collected from ILI cases identified by private practitioners were sent directly to the NIC during the entire period (1996–2007). The national postal service was responsible for the delivery of samples.

### Data management and analysis

Prior to 2007, data were entered by surveillance collaborators into an EpiInfo database (Version 6). Starting in 2007, a web-based integrated database (grippe.sante.gov.ma/INH) was used for data storage and sharing.

Influenza seasonality was compared with ILI and ARI temporal patterns from 1996 to 2009. Frequencies were calculated for demographics and clinical characteristics of patients with ILI and SARI with influenza during 2007–2009. During the 2008–2009 season, the monthly burden of outpatient consultations for influenza was estimated using data from public and private sentinel providers and the following formula:
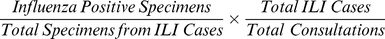



Interrupted Time Series Analysis (ITSA) was used to assess the impact (expressed as improvement of case detection) of the enhanced surveillance system introduced in 2007. For this, we used a segmented stepwise linear regression model that allows the partition of a time series into pre- and post-intervention segments [18], [19] using the year 2007 as the cut-off point. The constants (level) and slopes of the pre- and post-intervention regression models were used to assess the changes in performance of the system at the beginning of each period (constant) and the changes in trends over the two periods (slope). The following model was used for the analysis:
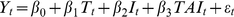
(1)Where:

- *Y_t_*: is the dependent variable in the model (number of samples)
- *β_0_*: estimates the baseline level of *Y_t_* at the beginning of the time series (the beginning of the influenza surveillance in Morocco in 1996)
- *β_1_*: estimates the trend of *Y_t_* during the pre-intervention period.
- *T_t_*: is a continuous variable indicating the time in years at time *t* from the start of the surveillance period.
- *β_2_*: estimates the change in baseline level after the intervention
- *I_t_*: is a dummy variable that defines the pre- (0) and post-intervention (1) period.
- *β_3_*: estimates the change in the slope from pre- to the post-intervention period
- TAI_t_: is a continuous variable indicating the number of years after the start of the intervention at time *t*.
- ε_t_: is the error term of the model.

Only predictors significant at α>0.05 were included in the final model. The analysis was implemented using SPSS 18.

## Results

### Influenza surveillance, 2007–2009

During the 2007–2008 and 2008–2009 seasons, 3102 specimens were collected from patients at sentinel sites. Of those, 2249 (73%) were from patients with ILI, and 853 (27%) were from patients with SARI. Among the ILI patients, 1371 (61%) were identified by the private sentinel network, and 878 (39%) by the public sentinel clinics. Patients 25–59 years of age accounted for the highest proportion of ILI cases during both seasons (44% during 2007–2008 and 39% during 2008–09). Among the SARI cases, children less than 5 years of age accounted for 86% of cases during 2007–2008 and 79% of cases during 2008–2009. Three percent (39/1400) of cases had received influenza vaccine during 2007–08, and 4% (66/1702) had received influenza vaccine during 2008–09 (Table 1).

**Table 1:** Demographic characteristics of influenza-like illness (ILI) and severe acute respiratory illness (SARI) patients, Morocco, 2007–2009.

Season	2007–08*	2008–09**
ILI surveillance	N = 997	N = 1252
	n (%)	n (%)
**Network**		
Private network	559 (40)	812 (48)
Health units network	438 (31)	440 (26)
**Age**		
<5 year	169 (17)	271 (22)
5–14 year	136 (14)	177 (14)
15–24 year	167 (17)	216 (17)
25–59 year	440 (44)	483 (39)
>60 year	85 (9)	105 (8)
**Gender**		
Male	488 (49)	589 (47)
**Influenza Vaccination**	30 (3)	53 (4)

SARI surveillance	N = 403	N = 450
	n (%)	n (%)
Hospitals network	403 (29)	450 (26)
**Age**		
<5 year	345 (86)	351 (78)
5–14 year	27 (7)	38 (8)
15–24 year	10 (2)	22 (5)
25–59 year	20 (5)	26 (6)
>60 year	1 (<1)	13 (3)
**Gender**		
Male	255 (63)	261 (58)
**Influenza Vaccination**	9 (2)	13 (3)

*from September 2007 to August 2008.

**from September 2008 to August 2009.

Of the 3102 enrolled patients, 98 (3%) had laboratory-confirmed influenza, of whom, 85 (87%) were ILI patients, and 13 (13%) were SARI patients. Among ILI patients, the proportion of laboratory-confirmed influenza cases was highest in children less than 5 years of age (3/169; 2% in 2007–2008 and 23/271; 9% in 2008–2009) and patients 25–59 years of age (8/440; 2% in 2007–2009 and 21/483; 4% in 2008–2009). All SARI patients with influenza were 14 years of age or younger (Table 2). The median length of time from symptom onset to testing was 3 days for SARI cases less than 14 years of age and 4 days for those 15 years of age and over during the 2007–08 season, and 5 days for both age groups during the 2008–09 season.

**Table 2:** Proportion of patients with laboratory-confirmed influenza by age group and surveillance network, Morocco, 2007–2009.

Season	2007–08*		2008–09**	
ILI surveillance	n/N (%)	95% CI (%)	n/N (%)	95% CI (%)
**Network**				
Private network	6/559(1.07)	0.39–2.32	64/812 (7.88)	6.12–9.95
Health units network	9/438 (2.05)	0.94–3.86	6/440 (1.36)	0.50–2.94
**Age**				
<5 year	3/169 (1.78)	0.37–5.09	23/271 (8,49)	5.46–12.46
5–14 year	2/136 (1,47)	0.18–5.21	10/177 (5,65)	2.74–10.14
15–24 year	1/167 (0,59)	0.00–3.29	12/216 (5,56)	2.90–9.50
25–59 year	8/440 (1.82)	0.79–3.55	21/483 (4,35)	2.71–6.57
>60 year	1/85 (1,18)	0.03–6.38	4/105 (3.81)	1.05–9.47

SARI surveillance	n/N (%)	95% CI (%)	n/N (%)	95% CI (%)
Hospitals network	4/403 (0.99)	0.27–2.52	9/450 (2.00)	0.92–3.76
**Age**				
<5 year	1/345 (0,29))	0.00–1.60	8/351 (2,28)	0.99–4.44
5–14 year	3/27 (11.11)	2.35–29.16	1/38 (2,63)	0.07–13.81
15–24 year	0/10 (0.00)	0.00–30.85	0/22 (0.00)	00.00–15.44
25–59 year	0/20 (0.00)	00.00–16.84	0/26 (0.00)	00.00–13.23
>60 year	0/1 (0.00)	0.00–97.50	0/13 (0.00)	00.00–24.70

*from September 2007 to August 2008.

**from September 2008 to August 2009.

### Influenza virus circulation and seasonality

During 2007–2008, influenza A(H1N1) (5/19; 26%) and B (14/19; 74%) (Yamagata and Victoria lineages) were detected from specimens taken from ILI and SARI patients; during 2008–2009, influenza A(H1N1) (39/79; 49%), A(H3N2) (30/79; 38%) and B (10/79; 13%) (Victoria lineage) were detected. Twenty-nine specimens tested positive for viruses other than influenza including: RSV (25/29; 86%), adenovirus (2/29; 7%), and parainfluenza viruses (2/29; 7%).

During the 2007–2008 influenza season, influenza viruses were isolated from week 49/07 (December 3–9, 2007) to week 13/08 (March 24–30, 2008) and peaked during weeks 3/08 and 4/08 (January 14–27, 2008). During the 2008–2009 influenza season, influenza viruses were detected from week 43/08 (October 20–26, 2008) to week 16/09 (April 13–19, 2009) and peaked during weeks 1/09 and 2/09 (December 29, 2008–January 11, 2009).

**Figure 1:**
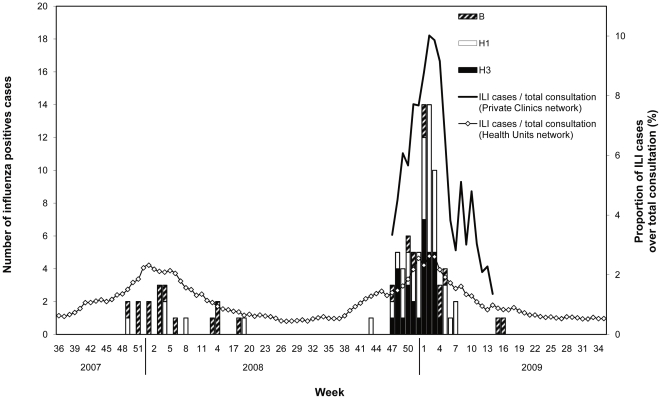
Number of positive influenza samples by type and subtypes and proportion of ILI cases over total consultation in the health unit and private clinic networks by week, Morocco, 2007–2009.

The total number of ILI cases and total number of consultation were available from the public health unit network for 2007–2009 and from the private network for 2008–2009. The proportion of ILI cases over total number of consultations in both the public health units and private networks increased concomitantly with the circulation of influenza viruses (Figure 1). During the 2008–2009 influenza season, the estimated monthly proportion of all patient consultations in the private network that was associated with laboratory-confirmed influenza began to increase during the month of November (0.6%) and peaked in January (1.3%). A similar pattern was observed in the public sector where the proportion of all patient consultations associated with laboratory-confirmed influenza began to increase in December (0.03%) and January (0.05%), peaked in February (0.1%), and declined to baseline levels in March (0%).

**Figure 2:**
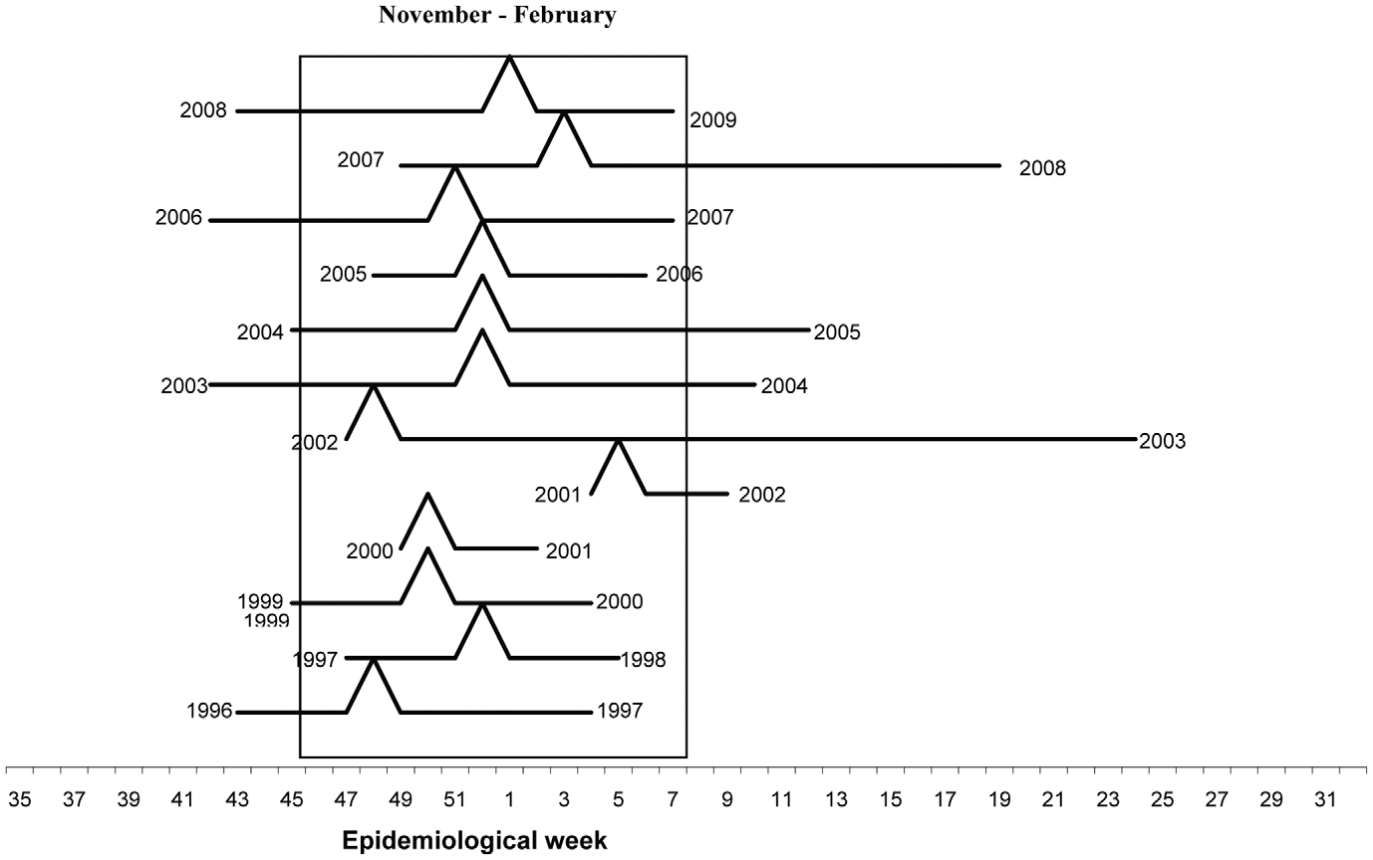
Onset peak and duration of influenza seasons, Morocco, 1996–2009.

During 1996–2007, the NIC received a total of 3363 specimens with a minimum of 94 specimens during the 2000–2001 season and a maximum of 634 specimens during the 2004–2005 season. During the entire surveillance period (1996–2009), influenza viruses were isolated from October to March, although the period of peak influenza virus circulation varied across seasons (Figure 2).

### Assessment of the enhanced surveillance system

**Figure 3:**
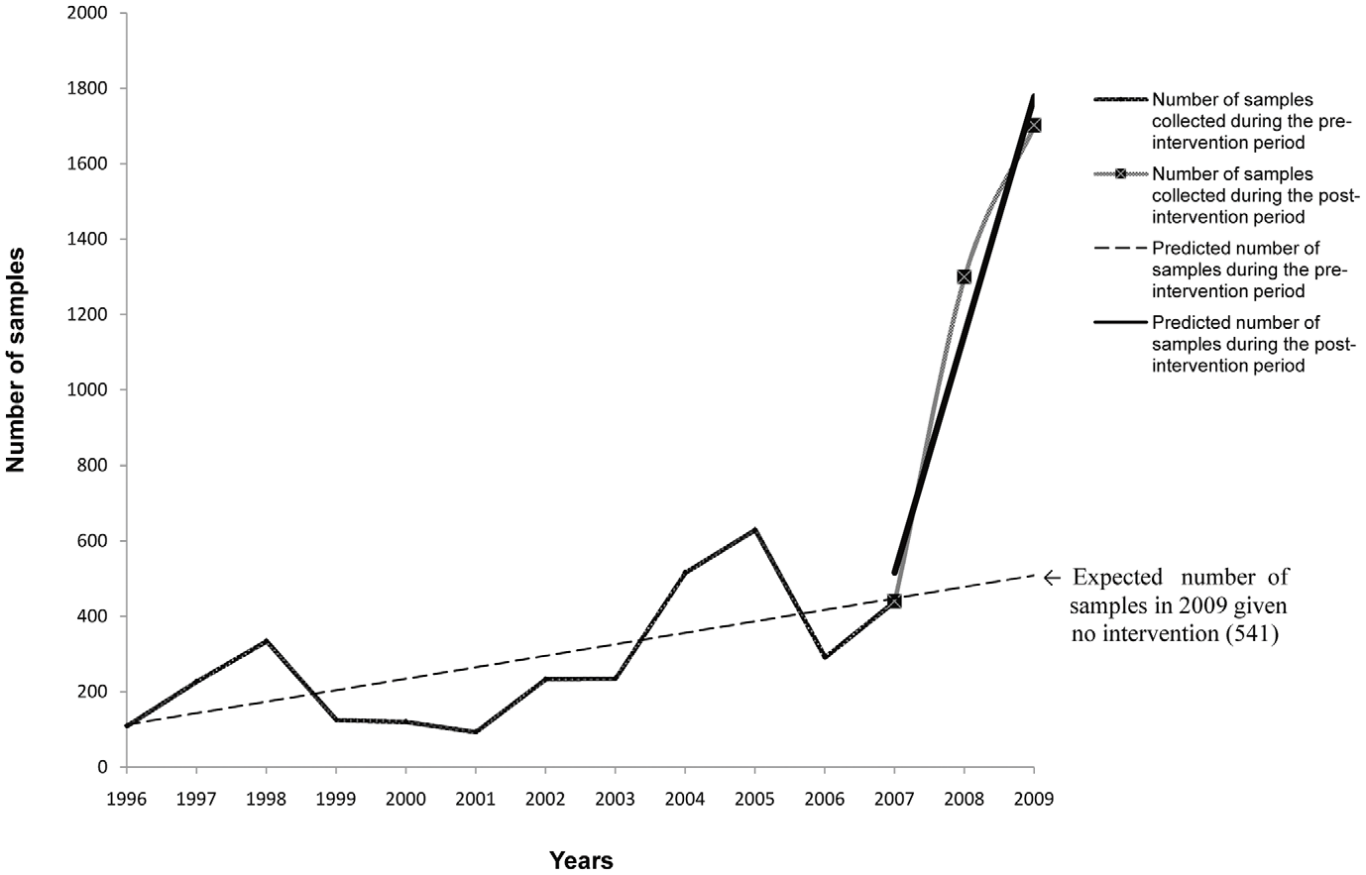
Observed and predicted (using the Interrupted Time Series Analysis) numbers of samples during the pre- and post-intervention period, Morocco, 1996–2009.

During 1996–2006, preceding the introduction of the enhanced influenza surveillance, the estimated average number of additional samples for each consecutive season was 33 (e.g. 33 more samples expected on average from one season to the next). After 2007 when the enhanced surveillance system was implemented, the estimated average number of additional samples for each consecutive season was 623 (Table 3). If the enhanced surveillance system had not been implemented, the expected total number of samples for the 2008/09 season would have been 541 (Figure 3). The 1702 samples collected in the 2008–2009 season represent a 215% increase in the total number of samples compared with the number expected in the same season if the surveillance system remained the same (541).

**Table 3:** Model coefficient, 95% confidence interval and p-value for the Interrupted Time-Series Regression Analysis for the assessment of the impact of the enhanced surveillance system, Morocco, 1996–2009.

Parameter	Coefficients	95% CI	p
Constant (at the beginning of the surveillance system: 1996)	68.1	−127.4–263.6	0.459
Time (during the pre-intervention period)*	33.8	7.5–59.9	0.016
Time (during the post-intervention period)**	623.4	434.0–812.7	<0.001

*the slope coefficient represent the expected average increase of number of samples in each consecutive year during the pre-intervention period (1996–2006).

**the slope coefficient represent the expected average increase of number of samples in each consecutive year during the post intervention period (2007–2009).

## Discussion

We described the epidemiology and seasonality of influenza in Morocco using influenza surveillance data from 1996 to 2009. We found that influenza viruses affected all age groups and circulated seasonally during the Moroccan winter months of October through April with peak circulation occurring frequently in December, similar to patterns documented in the temperate regions of the Northern hemisphere [10]–[13]. In addition we found that influenza accounted for 4%–9% of hospitalizations for SARI.

Children less than five years of age accounted for more than two thirds of all patients hospitalized with severe acute respiratory illness at sentinel sites in Morocco, and 1% of SARI patients in this age group had laboratory-confirmed influenza. Our findings contribute to the growing literature documenting that young children are at increased risk for severe respiratory infection, and that influenza contributes to the burden of severe respiratory infection in young children globally [20]–[22]. Both the burden of severe respiratory infection and the proportion due to viral etiologies including influenza are largely undocumented in Africa highlighting the need for continued development of respiratory illness surveillance in Africa. [14], [15]

Although influenza sentinel surveillance has been ongoing in Morocco since 1996, the enhancement of the surveillance system in 2007 to include public clinic networks and SARI surveillance substantially increased the number of respiratory specimens obtained from the system. In addition, the enhanced surveillance system permitted to estimate the rates of influenza infection requiring medical care and the burden of influenza on outpatient consultations and inpatient admissions.

Prior to 2007, the burden of influenza-associated consultations on private practitioners in Morocco was unknown. Through expansion of syndromic influenza surveillance to include the private sector in Morocco, we found that ILI and ARI consultations accounted for a larger proportion of all consultations seen in the private sector than in the public sector, highlighting the importance of engaging private practitioners in respiratory illness surveillance when feasible in communities that utilize private practitioners for acute medical care.

Despite the importance of these preliminary results, our surveillance system has limitations. Despite the increased number of samples obtained under the enhanced surveillance system, the rate of influenza virus detection remains low. The enhanced surveillance system in Morocco is relatively new; thus, participating clinicians may not have correctly identified all ILI or SARI cases, and specimen collection and storage techniques may not always have been optimal. In addition, the identification of influenza viruses was performed primarily using immunofluorescence assays which are less sensitive for the detection of influenza viruses than viral culture and real time reverse transcriptase polymerase chain reaction assays (rRT-PCR). The limited number of samples obtained during the initial years of surveillance hindered the use of time series analysis to statistically identify seasonal trends.

In conclusion, we document that influenza is responsible for both mild and severe respiratory illness in Morocco. A large majority of hospitalizations for SARI occur in children five years of age and under. During 1996–2009, the Moroccan influenza sentinel surveillance system generated important baseline data on respiratory illness and the proportion attributable to influenza; these data could be used to inform future evaluations of the impact of influenza prevention programs.
